# A Sudden Fluctuation in Creatinine Kinase: Water Intoxication and Rhabdomyolysis

**DOI:** 10.7759/cureus.10698

**Published:** 2020-09-28

**Authors:** Hiroshi Ito

**Affiliations:** 1 Hospital Medicine, University of Tsukuba Hospital, Tsukuba, JPN

**Keywords:** water intoxication, rhabdomyolysis, schizophrenia

## Abstract

Water intoxication often causes hyponatremia. Acute hyponatremia and its rapid correction have been reported to cause rhabdomyolysis. However, little is known about the clinical course of water-intoxication-related rhabdomyolysis. We report a case of self-induced water intoxication resulting in rhabdomyolysis, in which serum creatinine kinase surged rapidly. The appropriate selection of fluid therapy was difficult because of the differences in the standard treatments for each complication. Water restriction is used to treat water intoxication, while the opposite, fluid resuscitation, is used to treat rhabdomyolysis. Close monitoring of serum creatinine kinase was useful in determining fluid management in such a situation.

## Introduction

Self-induced water intoxication is common among psychiatric patients, affecting up to 20% of this patient population [1], and water intoxication often results in hyponatremia [2]. Rhabdomyolysis caused by acute hyponatremia and its rapid correction has been reported recently. However, little is known about the clinical course of water-intoxication-related rhabdomyolysis. In this report, we present a case of rhabdomyolysis with a sudden rise in serum creatinine kinase following self-induced water intoxication.

## Case presentation

A 44-year-old Japanese man with a history of schizophrenia was admitted to our hospital due to a 15-minute convulsion and subsequent impaired consciousness. His vital signs showed a heart rate of 80 beats per minute, a blood pressure of 117/99 mmHg, a temperature of 97.7 °F, oxygen saturation of 99% on room air, and a respiratory rate of 25 breaths per minute. A physical examination showed no remarkable changes. Laboratory tests revealed hyponatremia (110 mEq/L), hypokalemia (2.9 mEq/L), and an elevated serum creatinine kinase (1,579 U/L) (Table 1). Precontract CT of the head showed no remarkable findings.

**Table 1:** Laboratory test results of the patient’s blood samples After the patient’s serum sodium recovered on hospital day three, serum creatinine kinase surged to 47,900 U/L, peaking at 88,400 U/L on hospital day four

Variables	Day 1	Day 2	Day 3	Day 4	Day 5	Day 6	Day 9	Day 11
White blood cell (/μL)	17,500	7,800	6,300	6,500	5,300	4,200	4,600	3,900
Hemoglobin (g/dL)	12.1	12.2	12.5	12.1	12.6	11.9	12.7	12.7
Platelet (×10⁴/μL)	13.6	16.5	13.9	14.7	16.4	16.5	19.2	20.2
Aspartate aminotransferase (U/L)	39	57	486	479	696	552	100	44
Alanine aminotransferase (U/L)	21	21	57	65	106	114	70	47
Lactate dehydrogenase (U/L)	358	335	1,901	998	1,376	665	249	211
Sodium (mEq/L)	110	126	136	141	141	143	140	141
Chlorine (mEq/L)	76	91	102	106	104	105	102	102
Pottasium (mEq/L)	2.9	3.3	3.4	3.6	3.8	3.9	4.1	4.1
Urea nitrogen (mg/dL)	5.2	3.8	2.5	4.1	6	10.2	13	13.9
Creatinine (mg/dL)	0.57	0.6	0.6	0.58	0.59	0.62	0.66	0.67
Creatinine kinase (U/L)	1,579	4,896	88,400	55,506	65,320	39,153	2,544	890
C-reactive protein (mg/dL)	<0.03	0.73	3.38	2.07	1.08	0.81	0.3	0.09

**Figure 1:**
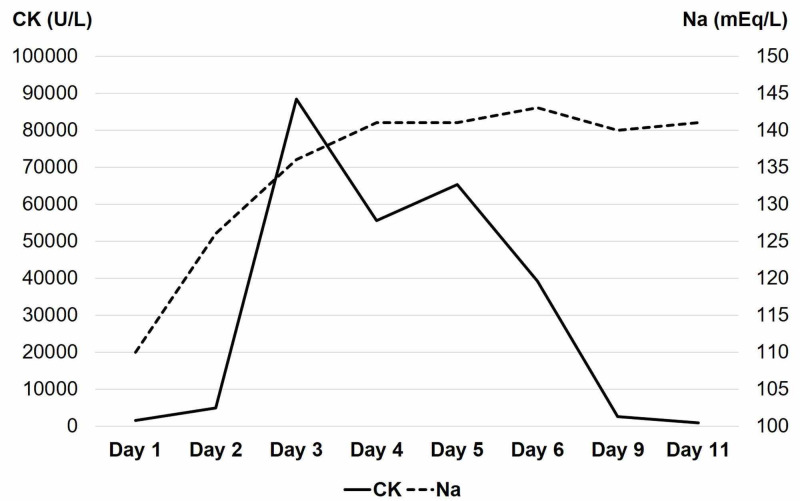
A time course of changes in the patient’s serum sodium and creatinine kinase After the correction of serum sodium, the patient’s creatinine kinase rose rapidly but dropped soon after administering acetate Ringer’s solution Na: serum sodium; CK: serum creatinine kinase

Although the exact amount was unclear, the patient tended to drink excessive amounts of water, according to his caregiver. Based on his medical history and laboratory findings, he was suspected of suffering from self-induced water intoxication. Acetate Ringer’s solution was initiated to correct hyponatremia. However, his urine output was approximately 10 L during the first 10 hours, and his serum sodium increased rapidly to 126 mEq/L on hospital day two. To relieve the overcorrection of serum sodium, he received 5% dextrose solution intravenously. On hospital day three, his serum sodium reached 136 mEq/L, and he regained consciousness. However, his serum creatinine kinase surged to 47,900 U/L, peaking at 88,400 U/L on hospital day four (Figure 1).

Acetate Ringer’s solution was initiated again, and his serum creatinine kinase dropped rapidly. A urinalysis on hospital day six showed no occult blood reaction, and his remaining clinical course was uneventful. He was then referred to a long-term hospital on hospital day 13.

## Discussion

We presented a case of water-intoxication-related rhabdomyolysis, in which a sudden rise and drop in serum creatinine kinase occurred. There have been several reports of water-intoxication-related rhabdomyolysis in patients with and without psychiatric disorders (Table 2) [3-9]. The underlying pathophysiology of this condition has not been elucidated, but one hypothesis is that increased intracellular calcium followed by decreased ion exchange could lead to muscle cell death. Another theory is that fluctuations in extracellular osmolarity could alter transmembrane potentials, resulting in muscle cell death [6]. Although the causes of water-intoxication-related rhabdomyolysis seem multifactorial, rapid correction of serum sodium has been proposed as a risk factor in several studies [10,11]. In our case, rhabdomyolysis was already evident on admission and was worsened by the rapid correction of serum sodium from 110 to 126 mEq/L during the first 10 hours.

**Table 2:** A summary of previous reports on water-intoxication-related rhabdomyolysis Na: serum sodium; CK: serum creatinine kinase; AKI: acute kidney injury

Author	Age of the patient	Gender	Underlying diseases	Initial Na (mEq/L)	Na correction rate (mEq/L/hr)	Maximal CK (U/L)	AKI	Prognosis
Ting JY [3]	41	Male	Schizophrenia	113	Not available	49,300	No	Survived
Strachan et al. [4]	63	Male	Bipolar disorder	110	0.38 (first 24 hours)	10,642	No	Survived
Katsarou et al. [5]	39	Male	Bipolar disorder	104	1.04 (first 24 hours)	16,339	Yes	Survived
Ulstrup et al. [6]	30	Male	Schizophrenia	115	0.78 (first 18 hours)	29,900	No	Survived
Dubin et al. [7]	58	Male	Schizophrenia	110	0.75 (first 24 hours)	26,760	No	Survived
Zaman et al. [8]	59	Male	Psychogenic polydipsia	111	0.27 (first 48 hours)	37,096	No	Survived
Fernando et al. [9]	41	Male	Ureteric calculus	119	0.71 (first 24 hours)	54,841	No	Survived
Our case	44	Male	Schizophrenia	110	0.67 (first 24 hours)	88,400	No	Survived

**Figure 2:**
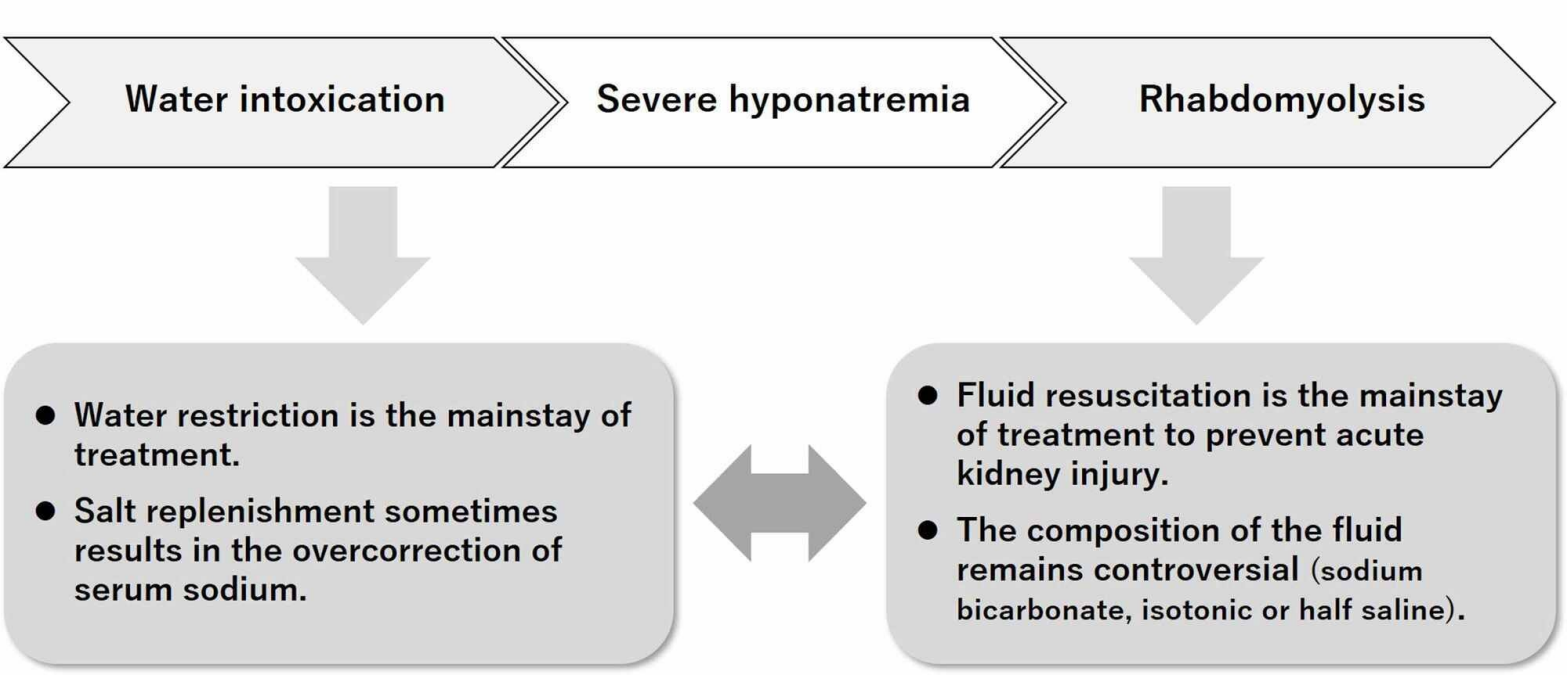
The dilemma in choosing fluid therapy for water-intoxication-related rhabdomyolysis Water restriction is the mainstay of treatment for hyponatremia secondary to water intoxication. Salt replenishment in this condition sometimes results in the overcorrection of serum sodium. In contrast, fluid resuscitation is the mainstay of treatment for rhabdomyolysis to prevent acute kidney injury

Appropriate intravenous rehydration is necessary to manage water-intoxication-related rhabdomyolysis. Hypertonic 3% saline is often used for severe hyponatremia [2], and aggressive fluid resuscitation is usually required to treat rhabdomyolysis [12]. However, the indication of hypertonic or isotonic saline administration should be carefully considered when treating water-intoxication-related rhabdomyolysis to avoid overcorrection of serum sodium. The secretion of antidiuretic hormone is physiologically suppressed in these patients, resulting in the rapid excretion of free water and an unexpected rise in serum sodium [1]. In our case, a balanced crystalloid solution was initiated on admission and administered continuously, which resulted in the overcorrection of serum sodium. We buffered this overcorrection on hospital day two using intravenous 5% dextrose solution. Nevertheless, the patient’s rhabdomyolysis worsened rapidly on hospital day three. The proper selection of fluid therapy seems essential in managing rhabdomyolysis secondary to self-induced water intoxication (Figure 2).

## Conclusions

Self-induced water intoxication can result in rhabdomyolysis, in which a rapid surge in serum creatinine kinase occurs. The selection of appropriate fluid therapy for water intoxication is complicated. However, selecting fluid therapy based on the patient’s current condition and adjusting it in response to subsequent changes in the patient’s condition seems essential for the management of water-intoxication-related rhabdomyolysis. Physicians should monitor serum creatinine kinase closely when they treat patients with hyponatremia secondary to water intoxication.
